# Accidents involving lithium-ion batteries in non-application stages: incident characteristics, environmental impacts, and response strategies

**DOI:** 10.1186/s13065-025-01445-x

**Published:** 2025-04-13

**Authors:** Ziyu Wang, Guohe Huang, Zhikun Chen, Chunjiang An

**Affiliations:** 1 Department of Building, Civil and Environmental Engineering, Concordia University, Montreal, QC H3G 1M8 Canada; 2 Institute for Energy, Environment and Sustainable Communities, University of Regina, Regina, SK S4S 0A2 Canada

**Keywords:** Lithium-ion battery, Thermal runaway, Environmental risk, Accident response, Standards

## Abstract

With the rapid growth of electric vehicle adoption, the demand for lithium-ion batteries has surged, highlighting the importance of understanding the associated risks, particularly in non-application stages such as transportation, storage, assembly, and disposal. This review explores the types and causes of lithium-ion battery accidents, categorizing them into leakage, fire, and explosion, often resulting from electrical, thermal, and mechanical abuses. It examines the environmental impacts of such incidents, including the release of toxic substances that threaten public health and ecological systems. The research also outlines the need for effective risk assessment methods and compliance with safety standards. Furthermore, it evaluates current emergency response strategies, advocating for a unified approach to managing these incidents. By delving into the complexities of lithium-ion battery safety, this study aims to contribute to improved practices and regulatory frameworks, ultimately enhancing related accident responses.

## Introduction

**Fig. 1:**
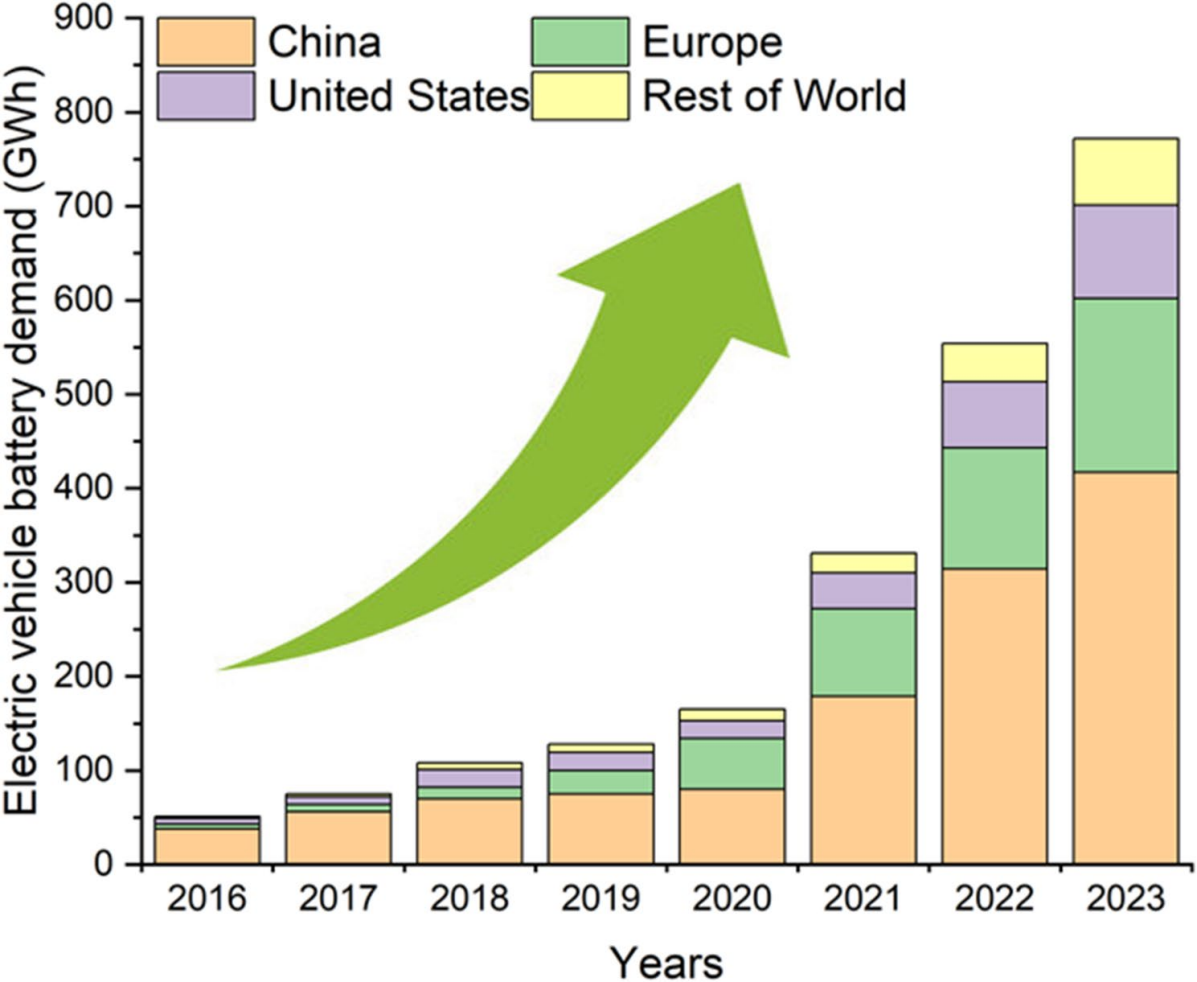
Electric vehicle battery demand by region from 2016 to 2023 [8]

With the advancement of society and technology, lithium-ion batteries are considered an important energy storage device for the future [1, 2]. Compared to other types of batteries, such as lead-acid batteries and nickel-cadmium batteries, lithium-ion batteries possess characteristics like high energy density, low self-discharge rate, and long lifespan [3–5]. Additionally, the growth of the electric vehicle (EV) market has driven the larger-scale application of automotive batteries, making lithium-ion batteries the preferred choice for EVs [6, 7]. As shown in Fig. 1, from 2016, when EVs began gaining traction in the personal vehicle market, global demand for EV batteries increased more than fifteen times (from 51 to 772 GWh) by 2023 [8]. Given this growing trend, it is expected that by 2030, the global demand for lithium-ion batteries will rise approximately twenty times from the 2020 level, reaching 2,500 GWh [9].

Despite their advantages and promising development prospects, lithium-ion batteries face safety challenges. Lithium-ion batteries exhibit good safety performance under normal conditions. However, when exposed to high-temperature pyrogens, subjected to external forces, or overcharged, the battery components may decompose and trigger exothermic reactions, eventually leading to thermal runaway (TR) [10, 11]. In the TR state, the electrochemical reaction of the battery involves not only the intercalation and deintercalation of Li^+^ between the positive and negative electrodes, but also the decomposition of the electrolyte interface film, the release of oxygen from the positive electrode, and other parasitic side reactions between the electrolyte and the electrodes [12, 13]. These reactions can increase the battery temperature, raise the risk of oxygen release from the active positive electrode material, and ultimately lead to battery rupture and explosion [14].

TR is often accompanied by the release of toxic gases, fires, and explosions in real-world accidents. In such cases, toxic substances and pollutants from lithium-ion batteries can spread in the form of liquids, particles, and gases into the air, water, and soil near the accident site [15, 16]. Among all toxic substances, hydrogen fluoride is the most concerning as it can cause corrosion of human tissue and systemic toxicity. Due to its chemical properties, it can enter the body through the skin and respiratory system and cause irreparable damage to organisms near the accident site at extremely low doses [17]. Other obvious problems include deterioration of air quality and acidification of soil and water bodies [18]. Current research focuses on accidents that occur during the use phase of lithium-ion batteries, but there is limited knowledge and understanding of the causes, impacts, and responses to accidents that occur in other stages of the lithium-ion battery life cycle [17, 19]. Recent incidents, such as the battery factory in Hwaseong, South Korea, serve as warnings to professionals and stakeholders that accidents involving lithium-ion batteries during the non-application stages pose increasingly serious risks as battery demand grows, life cycles lengthen, and supply chains become more complex.

This study first reports the types and causes of lithium-ion battery accidents in the non-application stages, which serves as an essential basis for the impact assessment and subsequent handling of the accidents. Next, the potential environmental impacts of accidents, along with the subsequent risk analysis and assessment, are discussed. Lithium-ion battery businesses, fire and environmental departments, and community residents need to understand this topic to improve existing battery design and risk preparation and reduce the frequency and severity of potential accidents. In Sect. 4, a response framework for fire departments and stakeholders to address accidents is developed to fill the relevant knowledge gap. In summary, the authors provide researchers and other stakeholders with a comprehensive knowledge framework on the characteristics, environmental impacts, impact assessment methods, and response strategies of accidents involving lithium-ion batteries in the non-application stages and make recommendations for future research.

## Incident types and causes

Among lithium-ion battery accidents, there are three main types: leakage, fire, and explosion. These incidents often do not occur alone; for example, leakage may cause subsequent fire and explosion. In addition, these incidents may occur at any of the four non-application stages of lithium-ion batteries, including transportation, storage, assembly and maintenance, and disposal. The transportation stage refers to the movement of lithium-ion batteries by truck, train, plane, and cargo ship to the next point in their life cycle [20]. Examples include transportation from battery production plants to EV assembly sites or from EV disassembly sites to landfills. Although transportation-related accidents are not the most frequent, their occurrence is increasing as supply chains become more complex [21]. The storage stage is particularly prone to lithium-ion battery accidents, mainly due to the uncertainty of the storage environment. In many cases, batteries are not classified as hazardous or special goods and are stored under the same conditions as more stable and safer products, without sufficient consideration of their specific requirements [22].

**Fig. 2:**
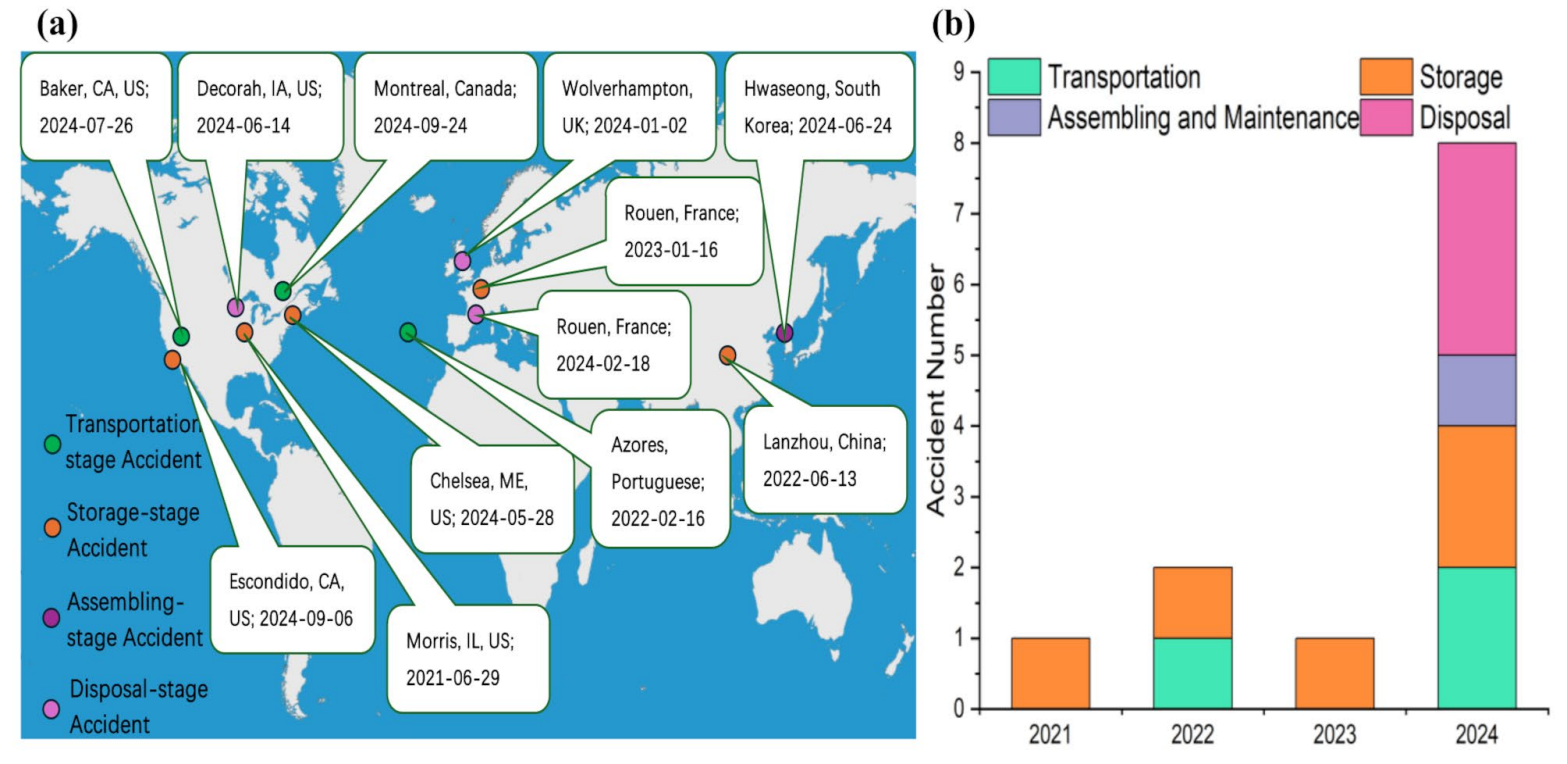
(**a**) Map of accidents in non-application stages of lithium-ion batteries worldwide since 2021; (**b**) the number of accidents occurring at different stages each year [28–39]

The assembly and maintenance stage is also experiencing rapid development for two reasons. First, the rise of battery manufacturing plants and new lithium-ion battery technologies has increased battery assembly activities worldwide [23, 24]. Second, some EV manufacturers are establishing battery swapping stations, allowing drivers to replace their car batteries without stopping to charge. The replaced batteries are subsequently left at the station for maintenance and charging. With more participants, emerging technologies, and increasingly complex supply chains, the likelihood of battery accidents rises [25]. Finally, the disposal stage mainly includes three processes: recycling, repurposing, and landfilling. As shown in Fig. 2, accidents related to recycling surged in 2024 due to an increase in battery recycling. Repurposing lithium-ion batteries remains a technology in its pilot phase and requires further improvements in safety [26, 27].

The causes of lithium-ion battery accidents in non-application stages can also be classified into four categories: electrical abuse (overcharging and short circuit), thermal abuse (exposure to high-temperature heat sources), mechanical abuse (vibration, collision, and extrusion), and other environmental factors (high external pressure, soaking, immersion of damp heat, and saline atmospheres) [40]. Under normal circumstances, a lithium-ion battery consists of a complete negative electrode (anode), a positive electrode (cathode), an electrolyte, and a separator immersed in the electrolyte. In this case, lithium exists only as ions in the liquid electrolyte. When the battery charges or discharges, Li^+^ moves between the two electrodes. The insertion reaction in a lithium-ion battery is a reversible process, referring to the insertion of Li^+^ into the constituent materials of the electrode [41].

Electrical abuse mainly occurs during the assembly and maintenance stages and during the assessment process in repurposing. Overcharging or short circuits are triggered when the charging process of lithium-ion batteries fails to stop before the voltage reaches its upper limit, overcharging the battery with energy. After overfilling, the electrolyte decomposes at the interface between the positive and negative electrodes and slowly raises the battery temperature. Subsequently, excessive Li^+^ is extracted from the positive electrode, destabilizing the material and causing it to release oxygen, while excessive Li^+^ from the positive electrode is deposited on the negative electrode to form lithium dendrites, ultimately causing the battery to overheat and rupture [14]. In battery systems, the main cause of overcharging is often a malfunction of the battery management system (BMS) or the use of improper charging or assessing equipment. In contrast, short circuits are usually caused by two reasons. First, lithium dendrites form on the surface of an electrode and come into contact with another electrode due to design defects or inadequate manufacturing processes. Second, improper battery management during transportation, storage, and recycling leads to contact with other batteries, charged devices, or conductive materials [42, 43]. Thermal abuse may occur during transportation, storage, and disposal stages. The sources of heat include cigarette butts from staff, concentrated sunlight, or the spread of fire from other accidents. At high temperatures, due to the narrow temperature tolerance range of lithium-ion batteries, the decomposition of battery components and exothermic side reactions may occur inside the battery, which may cause fire and gas emissions but usually not cause an explosion [44].

Mechanical abuse mainly occurs during transportation, particularly in land and water transport collisions. For example, rough road surfaces can subject the EV battery packs to vibration loads. When the transport vehicle collides, the battery pack may be invaded by foreign objects such as structural parts of the transport vehicle’s body, inducing TR [45]. Water transport poses even higher risks due to increased vibration, humidity, and collision potential. Among them, the lack of moisture-proof measures and excessively high ambient humidity can cause corrosion of the battery shell or components and short circuits during transportation and storage, making accidents more likely to occur due to static electricity occurrence or proximity to heat sources [46, 47]. According to Zhang et al. (2023), high-humidity environments increase the likelihood of accidents involving lithium-ion batteries during sea transportation by 4.7% [48]. The consequences of accidents caused by mechanical abuse are often unpredictable due to factors like the direction of the applied force, battery pack arrangement, and battery housing design [49]. Accidents caused by environmental factors can also occur at any non-application stage, making it essential for stakeholders to maintain environmental control throughout the life cycle of lithium-ion batteries. For instance, long-term air transportation and storage at high altitudes can subject EV battery packs to external pressure, affecting their sealing and integrity and eventually causing accidents [22].

**Fig. 3:**
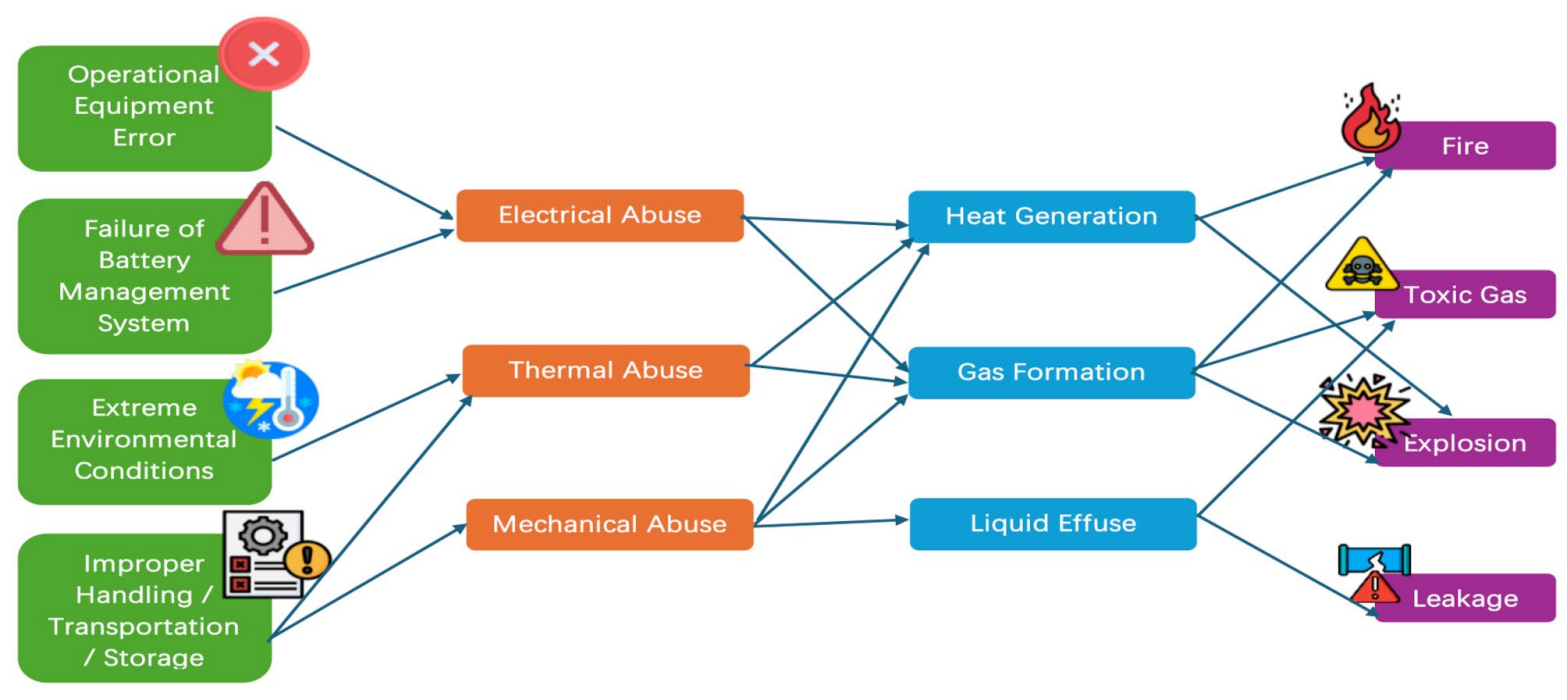
Schematic diagram of lithium-ion battery accidents caused by different factors

As mentioned earlier, the types and causes of lithium-ion battery accidents often do not occur alone. An example of a lithium-ion battery TR accident caused by multiple factors is as follows. After the onset of TR, smoke is released through cracks in the battery housing caused by mechanical abuse. The smoke consists of flammable and toxic gases, such as methane (CH_4_), carbon monoxide (CO), and hydrogen fluoride (HF). CO, the primary flammable and highly toxic gas produced in accidents, is generated by the insertion of lithium in the negative electrode, resulting in the reduction of CO_2_ and electrolytes. CH_4_ is generated by reducing electrolyte to lithium carbonate in a hydrogen environment when an accident occurs [50]. The details of HF will be discussed in the next section. A nearby heat source may ignite the flammable gases, causing flames to further heat the battery. If the released gas accumulates in an enclosed area and mixes with O_2_, an explosion may occur [51]. Figure 3 shows a schematic diagram of lithium-ion battery accidents caused by different factors.

## Environmental impacts and risk assessment

The impacts of incidents involving lithium-ion batteries primarily focus on fires and the release of toxic substances. In addition to threatening the safety and health of first responders and nearby residents, battery incidents can have broader environmental impacts. In such cases, the main channels of environmental impact are air, soil, and water. HF is of greatest concern because it can enter the human body through the skin or respiratory system, and the ingestion of even a few ppm causes severe corrosive effects and systemic toxicity [17]. There are two main ways that HF ​​is formed in lithium batteries. First, HF is directly generated through gassing reactions during charging and cycling. Second, when an accident occurs, LiPF_6_ in the electrolyte and its decomposition product PF_5_ react with humid air and decompose to form HF [52]. Dust, especially particles with an aerodynamic diameter of less than 10 μm (PM_10_) or 2.5 μm (PM_2.5_), is another atmospheric emission from battery accidents. These particles, when suspended in the atmosphere, can harm human health, negatively impact air quality, and reduce visibility [53]. Due to the composition of the battery, particles released through fire or explosion often combine with heavy metals, leading to more serious health problems for residents, such as cardiovascular disease. Once deposited, these particles may react with the surrounding water and soil, eventually threatening the local ecology [54, 55].

**Fig. 4:**
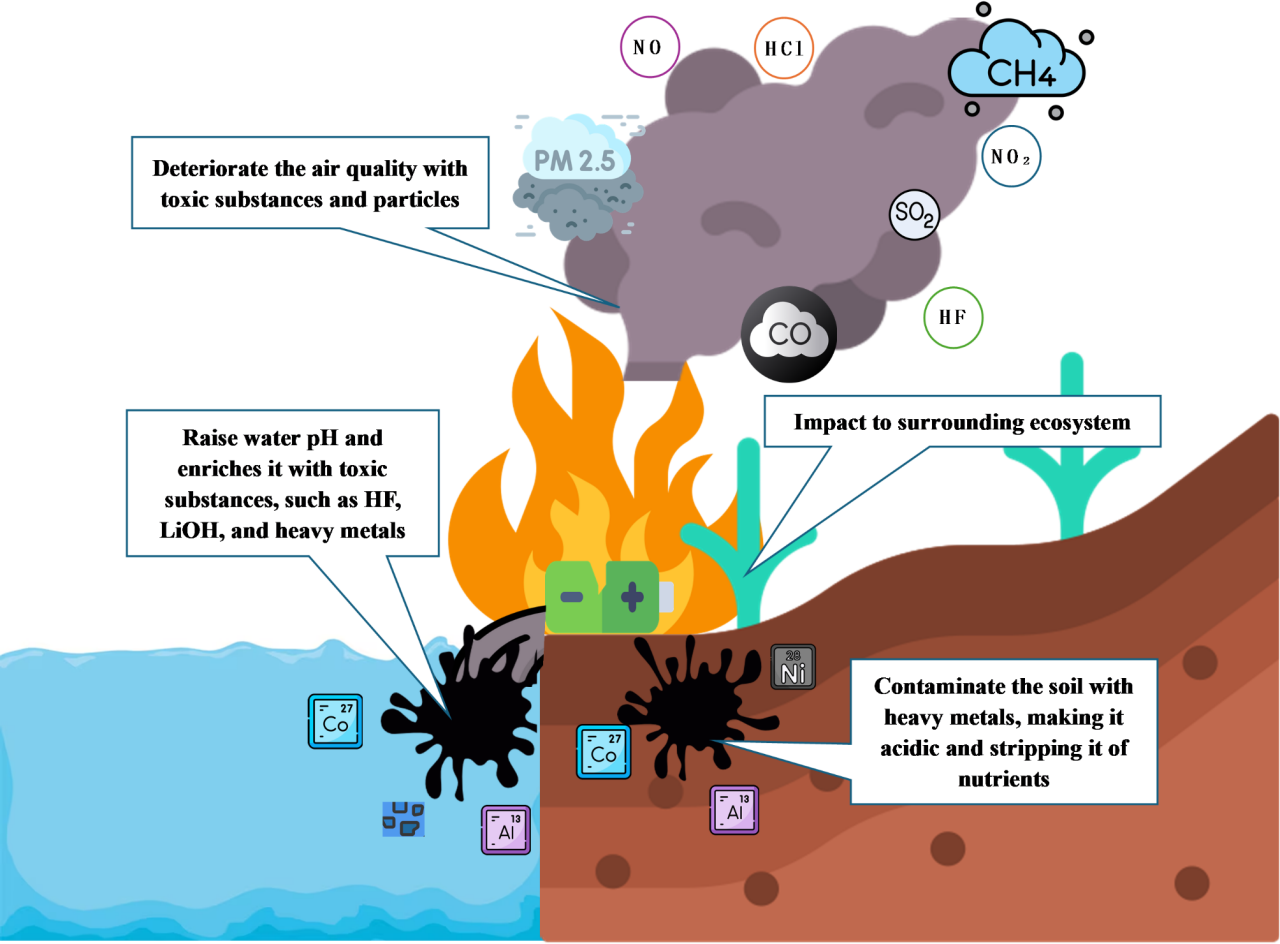
Pollution and risks caused by lithium-ion battery incidents

At the same time, other substances in air emissions, such as nitrogen dioxide (NO_2_), sulphur dioxide (SO_2_), and hydrogen chloride (HCl), can deteriorate air quality near the accident site. In the research conducted by Bugryniec et al. (2024), they reviewed 60 research articles related to gas production from lithium-ion battery TR failure. Based on the research of Wang et al. (2019), the amount of accident gas produced by different types of fully charged 18,650 lithium-ion batteries ranges from 76.5 to 265 mmol [50]. However, due to the diversity of battery designs and types and the rapid development of related technologies, there are still gaps in understanding the generation and emission of toxic and flammable gases that need to be addressed [51]. In addition to being present in air emissions, the pollutants and harmful substances mentioned above can also appear in liquid form and threaten nearby soil and water bodies in some accidents without fire or explosion, such as battery electrolyte leakage in landfills [18, 56]. Heavy metals, electrolyte degradation products, and dissolved toxic gases in battery leakage fluid have direct toxic effects and alter soil properties. These corrosive substances can lower the soil pH and deplete essential nutrients [57]. They also enhance the release of heavy metals in the soil, increasing the toxicity of these heavy metals under more acidic conditions [58]. Figure 4 illustrates the pollution and risks associated with lithium-ion battery incidents.

As stakeholders are concerned about the depletion of essential metal resources, such as nickel, cobalt, and lithium, used in the manufacture of lithium-ion batteries, recycling lithium-ion batteries has become an increasingly important component of the disposal stage. In industry, the two most commonly used recycling methods are pyrometallurgical and hydrometallurgical recycling [59]. Pyrometallurgical recycling processes large quantities of batteries and is not specific to a particular type of lithium-ion battery. However, its recycling efficiency is inferior to that of hydrometallurgical recycling technology, and it often fails to recover lithium metal [60]. The recovered products must be further processed, and the entire process can produce a significant amount of dust and residues [61]. The main causes of accidents in pyrometallurgical facilities are usually imperfect pretreatment and improper storage of waste batteries. The risk of accidents increases significantly when unsuitable recycled materials are exposed to high-temperature sources [18]. Hydrometallurgical recycling involves chemical treatment with inorganic and organic solvents. Although it can effectively recover all important metals in lithium batteries, it requires unique solutions for different types of lithium batteries, cannot process large quantities of batteries, and has wastewater treatment problems [62]. According to Kim et al. (2024), acid and alkali solutions used for metal purification, such as lithium hydroxide (LiOH), pose constant threats to workers in the facility, surrounding water and soil, and nearby residents if not used or disposed of properly [63]. When fires and explosions occur at hydrometallurgical recovery facilities, these chemicals can also cause additional problems, such as soil and water acidification.

In order to accurately assess the risk of accidents involving lithium-ion batteries in their non-application stages, it is crucial to have an accurate and comprehensive understanding of the characteristics of the relevant lithium-ion batteries [64]. The first step is to ensure that the battery in the accident complies with international and local safety and environmental standards and regulations. These standards are typically established by international organizations (the International Electrotechnical Commission (IEC), the International Organization for Standardization (ISO), and Underwriters Laboratories (UL)), as well as by national governments and professional associations like the National Fire Protection Association (NFPA) and the Chinese standard GB/T [65]. Table 1 outlines the various lithium-ion battery safety and environmental standards and regulations that are relevant to the scope of this article [66, 67]. According to the information in the table, it requires significant effort for a battery manufacturer to complete the entire life cycle of a battery in different places around the world [68]. In other words, when entering jurisdictions with different standards, lithium-ion batteries need to be re-certified and may require design adjustments. The relevant information gaps and changes undoubtedly increase the risk of accidents. At the same time, some tests and declaration processes in the standards are optional, which complicates accident analysis and evaluation. In addition, environmental standards specifically for non-application stages of lithium-ion batteries currently only target recycling in most parts of the world [69, 70]. For other processes, local environmental laws and regulations usually only involve typical pollutant emissions and wastewater and solid waste treatment [71–74]. Under some conditions, some environmental risks may occur; for example, hazardous substances, such as HF, which are not subject to environmental assessment or supervision, may enter the air and spread.

**Table 1:** International safety standards for lithium-ion batteries

Standard	Standard Name	Description
IEC 62,660	Secondary lithium-ion cells for the propulsion of electric road vehicles	The standard includes performance testing and reliability and abuse testing.
ISO 12,405	Electrically propelled road vehicles-Test specification for lithium-ion traction battery packs and systems	It includes high-power and high-energy applications and safety performance requirements.
UL 1642	Certification of Lithium-ion Battery	It is made to avoid the risk of injury to people due to fire or explosion.
UL 2580	Batteries for use in electric vehicles	It mainly evaluates the reliability of battery abuse and the ability to protect people.
GB/T 31,485	Safety requirements and test methods for traction battery of the electric vehicle	The objective is to outline the safety requirements and test methods for the battery cell.
NFPA 1	Fire Code	Minimum requirements to establish life safety and property protection from the hazards of fire and explosion.
NFPA 68	Standard on explosion protection by deflagration venting	Design to ensure that structural and mechanical damage is minimized.

To fully understand the causes of accidents and reduce the risks of the aforementioned issues, it is also essential to understand and improve the internal design of the battery. In battery accidents, the main contributors to risk are the cathode materials, electrolytes, and separators [75]. Cathode materials must maintain structural and chemical stability [76]. In the current battery market, the most stable cathode material is LiFePO_4_; however, the safety of cathode materials can be further improved. Coating, elemental doping, electrolyte film-forming additives, and high-voltage electrolyte additives are common methods to improve cathode safety [14]. The separator of a lithium-ion battery is a porous polymer film that separates the cathode from the anode and allows only ions to pass through. The commonly used polyolefin separator is fragile and easily shrinks at elevated temperatures. Improvements using ceramic particles or nanomaterials are feasible. The electrolyte is prone to combustion and explosion due to its chemical properties. Wu et al. (2021) developed an electrolyte synthesized from symmetrical tetra-butylphosphonium cations and imide anions, which can increase the stability of the positive electrode reaction of traditional lithium-ion batteries while improving the discharge capacity [77]. In addition, all-solid-state batteries are a potential research direction for future safe batteries because they have no separators and no liquid [78].

The second step is to determine the hazard intensity of the accident. For lithium-ion battery fires, the Heat Release Rate (HRR) is a parameter that can accurately assess the fire intensity [79, 80]. Although there are currently no established severity levels and definitions for lithium-ion battery accidents in non-application stages, Table 2 presents a modified list of hazard levels based on relevant standards for battery energy storage systems [14]. With the rapid development of artificial intelligence and machine learning technologies, recent studies have shown that combining intelligent algorithms with BMS systems can enable TR and fault detection of lithium batteries in their early and non-application stages, thereby improving safety [81, 82]. For example, Daniels et al. (2024) developed a random forest classification model to predict the location of TR batteries in battery modules based on temperature distribution data obtained from optimized temperature sensors for precise handling [83]. Another example is Das Goswami et al. (2024), who developed a framework that integrates the graph neural network and the long short-term memory network to identify potential fault hotspots and detect TR before it occurs [84]. It is foreseeable that with the advancement of battery design and intelligent algorithms, more powerful, innovative, and accurate TR and accident prediction tools will continue to emerge.

**Table 2:** Hazard severity levels for accidents in non-application stages of lithium-ion batteries

Hazard Severity Level	Description	Classification Criteria
0	No Effect	No effect, no loss of functionality.
1	Reversible Function Loss	No damage or hazard, reversible loss of function.
2	Damage	No hazard. Irreversible loss of function. Replacement or repair needed.
3	Minor Leakage	Evidence of cell leakage or venting with weight loss < 50% of electrolyte weight.
4	Major Leakage	Evidence of cell leakage or venting with weight loss > 50% of electrolyte weight.
5	Rupture	Loss of mechanical integrity of the container, resulting in release of contents.
6	Fire	Ignition combustion of flammable gas or liquid for more than one second.
7	Explosion	Very fast release of energy sufficient that may cause considerable structural bodily damage.

The third step is to assess the impact of the accident on the health of nearby residents and accident response participants, and the surrounding air, water, and soil. The health risk assessment primarily focuses on HF and LiOH produced by the reaction of lithium and water [85]. HF is a colorless gas with a strong irritating odor, toxicity, and strong corrosiveness, and LiOH is highly toxic [17]. The formation of LiOH primarily occurs due to the hydrophilicity of Li^+^, so when exposed to a water vapor environment, Li^+^ reacts with OH^−^ to form LiOH [86]. When determining the measurement range of HF and LiOH concentrations in the air, diffusion models, such as the Gaussian plume equation, can be used. By measuring wind speed, wind direction, and horizontal and vertical diffusion coefficients of pollutants and integrating the locations of major residential areas, the approximate assessment range can be determined [87]. After measuring the concentrations of HF and LiOH in the air and smoke, it is necessary to determine the inhalation exposure dose and the whole-body dose from transdermal exposure. The two results are then combined to obtain the margin of exposure (MOE) and compared with the standard values to complete the health risk assessment [88, 89]. In this case, areas where the MOE values ​​of pollutants exceed the standard MOE values ​​ require further emergency treatment. For the risk assessment of the surrounding environment, professionals need to sample and analyze the air, water, and soil near the accident site, comparing the results with local or international standards. Parameters like heavy metal content, particulate matter levels, pH value, and ecotoxicity require particular attention [90, 91].

## Accident response

Compared to the extensive research on battery TR, internal reactions, and materials, far fewer studies address battery accident response and related technologies [92, 93]. As mentioned earlier, lithium-ion battery accidents can vary due to different causes. The response types can be categorized as leakage, gas emission, and fire. Explosion is often the final result after a prolonged period of TR, so it is not considered a separate response category. The leakage of electrolytes and other toxic substances from the battery often leads to acidification and contamination of nearby water bodies and soil. Some potential response strategies include isolating the affected land and marking it as a brownfield, excavating contaminated soil and backfilling it, and using neutralizers and detoxifiers to remediate contaminated soil and water bodies [94, 95]. Gas emissions are easier to deal with than fire because gas emissions indicate that the fire has not yet spread or TR is still in its early stage. In such cases, water, water-based inhibitors or other cooling measures need to be applied to the battery immediately from a distance while ensuring good ventilation and limiting the spread of the fire [96]. It is worth noting that due to the presence of toxic and corrosive gases, all personnel close to the accident site need to use respiratory protection and remove their clothes and shower promptly after leaving the accident site. If managed promptly, battery gas emission accidents typically cause no damage beyond atmospheric emissions, though caution is required to prevent sudden explosions during cooling. The next step is to decontaminate the air and warn or evacuate the surrounding residents. Due to the complexity of accident sites and the composition of gases, the current solution for managing toxic gases primarily involves strengthening ventilation at the accident site to reduce the concentration of toxic gases as soon as possible or use water sprays to dissolve substances, such as HF, in water and promptly collect and dispose of on-site wastewater.

Emergency response procedures for lithium-ion battery fire accidents are the most complex for three main reasons. First, according to the five fire classes described in NFPA 10-2018, lithium-ion batteries involve four of them: class A (battery polymer separator), class B (liquid electrolyte), class C (other electronic components), and class D (lithium metal particles) [92]. Each fire class requires different suppressants, meaning a wide range of fire suppressants are theoretically available for lithium-ion battery fires, including dry powder, alkane, carbon dioxide, and water [97]. However, the second challenge is cooling, as high temperatures can cause battery fires to reignite. These reignition accidents are difficult to manage because they can occur randomly, even a few months after the initial fire [98]. Li et al. (2019) analogized the mechanism and required conditions of lithium-ion batteries reigniting the combustion of internal combustion engines based on the theory of internal combustion engine combustion, providing a reference and a new direction for subsequent research [99]. Two common strategies to prevent reignition are using large amounts of water-based fire suppressants for cooling and allowing the battery pack to burn out completely [100]. In other words, the reignition characteristics of lithium-ion battery incidents make dry powder, alkane, and carbon dioxide less appropriate. The last challenge relates to the disadvantages of water-based fire suppressants. Pollutants, such as HF and heavy metals, from lithium batteries may enter the surrounding environment with the water used for firefighting, posing secondary environmental risks [101]. According to Larsson et al. (2017), 20 to 200 mg/Wh of HF is generated by burning batteries [102]. When these water-soluble substances mix with fire extinguishing agents and flow into nearby soil and water bodies, they may cause more significant environmental damage than the diffusion of HF in the air and subsequent deposition in water bodies. Therefore, on-site collection and harmless treatment of wastewater must be considered. Furthermore, the fire-extinguishing efficiency of water-based suppressants is often slow, allowing the fire to spread or potentially cause an explosion [97]. Recent research has focused on combining water with additives to reduce the amount of water required for lithium-ion battery incidents, shorten fire extinguishing time, and prevent reignition. However, no ideal solution has been found to address the uniformity and high cost of additives [22, 103].

In daily life, the threat and risk of accidents caused by lithium batteries in non-application stages are often much lower than in commercial and industrial contexts. However, the relevant mechanisms and impacts still exist. In this case, one should call the fire alarm immediately to seek professional help. It is helpful to remove flammable and combustible materials near the fire point as far as possible while ensuring safety. If a large number of lithium batteries or products containing lithium batteries are stored at home, they should be stored following the guidance of manufacturers or professionals and equipped with appropriate fire extinguishing equipment.

**Fig. 5:**
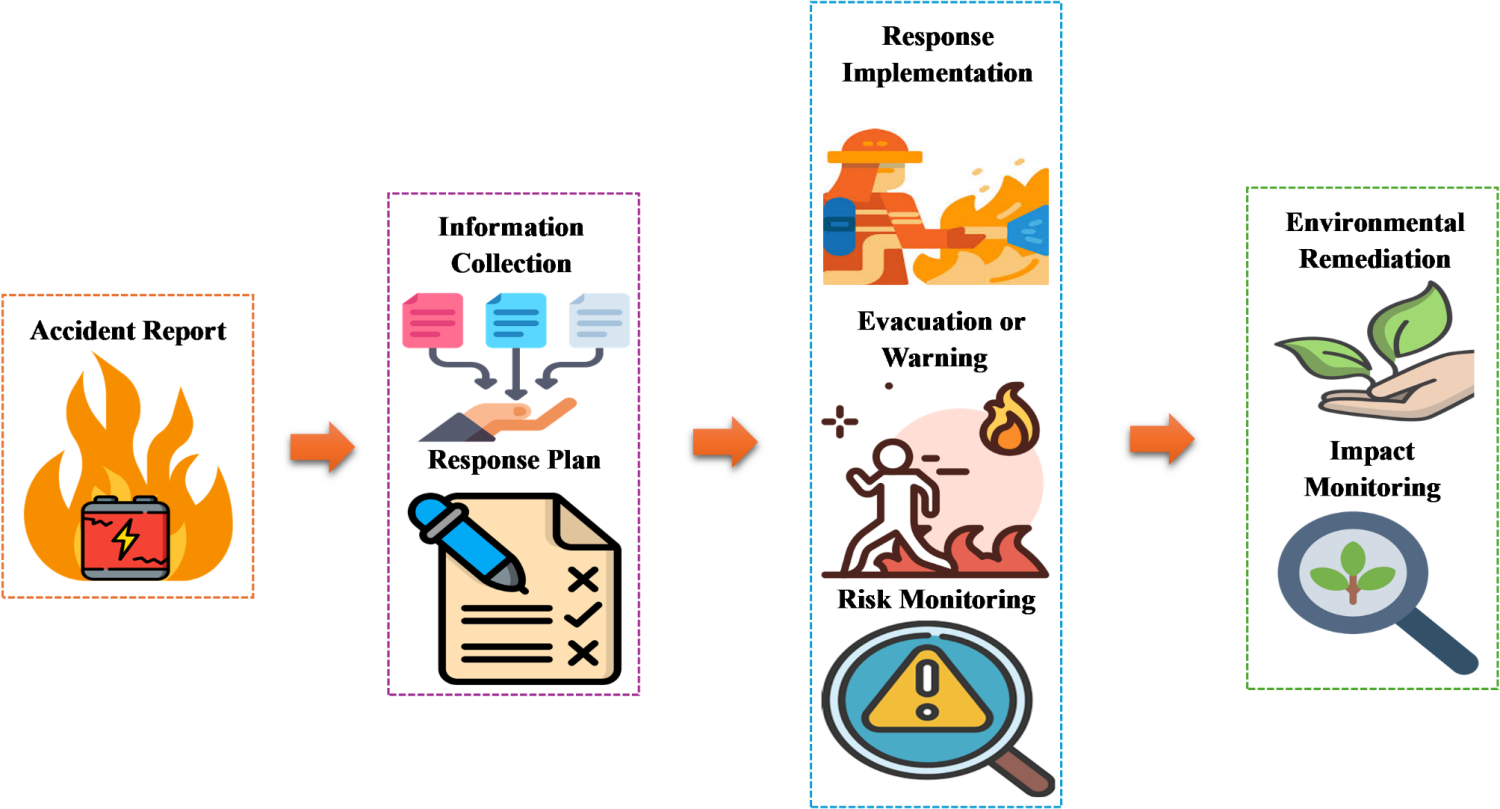
Response process of accidents in non-application stages of lithium-ion batteries

Since local fire and environmental departments typically handle these accidents, there is still a lack of unified and comprehensive response process [104]. This means that the response strategy often depends on the on-site commander. However, based on the previous descriptions, the general process of lithium-ion battery accident response can be summarized as shown in Fig. 5. When the accident is reported, the response unit in charge needs to immediately gather sufficient information from the site, the battery manufacturer, and the responsible party at the time to complete the initial response plan [105]. For instance, due to the diversity of accident causes and battery designs, the impact of the timing of the initial accident and the temperature on the probability of reignition is challenging to quantify; in this case, the fire department must work closely with the battery designer to find the optimal solution to prevent reignition. After implementing the response, it is necessary to evacuate nearby residents or issue warnings based on the on-site situation. Continuous risk monitoring is required at this stage to prevent sudden disasters or allow for adjustments to the response strategy as needed. After the accident is resolved, the surrounding environment should be restored, and long-term monitoring should be conducted to assess the effectiveness of recovery efforts.

## Recommendation and future research

Further research is needed on the potential risks and accident response of lithium-ion batteries in the non-application stages. The challenges and suggestions for future research are as follows:

(1) Follow up on new methods and systems for the production, transportation, maintenance, and disposal of lithium-ion batteries, such as replacing conventional charging with vehicle battery swapping. When new life cycle links emerge, new risks and potential accidents may also arise. In this case, targeted analysis and evaluation must be carried out promptly.

(2) Continue to pay attention to the working principles of new battery designs, especially all-solid-state lithium batteries, and the environmental and safety impacts of potential accidents. It is undeniable that different types of batteries perform differently when exposed to electrical, thermal, and mechanical abuse.

(3) Use machine learning and artificial intelligence technologies not only for risk prediction but also for accident impact assessment and accident response. These technologies can significantly increase the efficiency of assessment and response and reduce casualties and impacts caused by accidents.

(4) Develop response equipment for lithium-ion battery accidents, such as tools that can accurately and sensitively detect HF and LiOH concentrations at the accident site. In this case, targeted and effective response plans can be made more quickly.

(5) Develop standards and guidelines specifically for responding to lithium-ion battery accidents. As the number of accidents increases, targeted documents can better guide fire departments, stakeholders, and nearby residents in making more appropriate judgments and behaviors.

## Conclusions

In conclusion, this research highlights the multifaceted risks associated with lithium-ion battery accidents in non-application stages, including transportation, storage, assembly, and disposal. The categorization of incidents, such as leakage, fire, and explosion, reveals the interconnected nature of these risks, often precipitated by electrical, thermal, and mechanical abuse. The environmental consequences of such accidents, particularly the release of toxic substances and pollutants, pose significant threats to public health and ecosystems. Effective risk assessment methodologies are crucial for mitigating these hazards, necessitating compliance with established safety standards and proactive environmental monitoring. Moreover, the analysis of current emergency response strategies underscores the urgent need for a standardized and coordinated approach to managing lithium-ion battery incidents. By enhancing preparedness, improving response technologies, and fostering collaboration among stakeholders, the challenges posed by incidents in non-application stages of lithium-ion batteries can be more effectively addressed.

## Data Availability

No datasets were generated or analysed during the current study.

## Aabbreviations

BMS: Battery management system
CH_4_: Methane
CO: Carbon monoxide
CO_2_: Carbon dioxide
EV: Electric vehicle
GB/T: National Standards of the People’s Republic of China
HCl: Hydrogen chloride
HF: Hydrogen fluoride
HRR: Heat release rate
IEC: International Electrotechnical Commission
ISO: International Organization for Standardization
LiFePO_4_: Lithium iron phosphate
LiOH: Lthium hydroxide
LiPF_6_: Lithium hexafluorophosphate
MOE: Margin of exposure
NFPA: National Fire Protection Association
NO_2_: Nitrogen dioxide
O_2_: Oxygen
PF_5_: Phosphorus pentafluoride
PM_2.5_: Particles with an aerodynamic diameter of less than 2.5 μm
PM_10_: Particles with an aerodynamic diameter of less than 10 μm
SO_2_: Sulphur dioxide
TR: Thermal runaway
UL: Underwriters Laboratories
